# Technology engagement is associated with higher perceived physical well-being in stroke patients prescribed smartwatches for atrial fibrillation detection

**DOI:** 10.3389/fdgth.2023.1243959

**Published:** 2023-12-06

**Authors:** Edith Mensah Otabil, Qiying Dai, Paula Anzenberg, Andreas Filippaios, Eric Ding, Jordy Mehawej, Joanne E. Mathew, Darleen Lessard, Ziyue Wang, Kamran Noorishirazi, Alexander Hamel, Tenes Paul, Danielle DiMezza, Dong Han, Fahimeh Mohagheghian, Apurv Soni, Honghuang Lin, Bruce Barton, Jane Saczynski, Ki H. Chon, Khanh-Van Tran, David D. McManus

**Affiliations:** ^1^ Division of Cardiovascular Medicine, Department of Medicine, University of Massachusetts Chan Medical School, Worcester, MA, United States; ^2^ Division of Cardiovascular Medicine, Department of Medicine, Saint Vincent Hospital, Worcester, MA, United States; ^3^ Department of Internal Medicine, Central Michigan University, Mount Pleasant, MI, United States; ^4^ Department of Population and Quantitative Health Sciences, University of Massachusetts Chan Medical School, Worcester, MA, United States; ^5^ Department of Biomedical Engineering, University of Connecticut, Storrs, CT, United States; ^6^ Department of Pharmacy and Health Systems Sciences, School of Pharmacy, Northeastern University, Boston, MA, United States

**Keywords:** atrial fibrillation detection, smartwatch, Pulsewatch, self-reported physical health, anxiety, patient activation, self-reported mental health

## Abstract

**Background:**

Increasing ownership of smartphones among Americans provides an opportunity to use these technologies to manage medical conditions. We examine the influence of baseline smartwatch ownership on changes in self-reported anxiety, patient engagement, and health-related quality of life when prescribed smartwatch for AF detection.

**Method:**

We performed a *post-hoc* secondary analysis of the Pulsewatch study (NCT03761394), a clinical trial in which 120 participants were randomized to receive a smartwatch-smartphone app dyad and ECG patch monitor compared to an ECG patch monitor alone to establish the accuracy of the smartwatch-smartphone app dyad for detection of AF. At baseline, 14 days, and 44 days, participants completed the Generalized Anxiety Disorder-7 survey, the Health Survey SF-12, and the Consumer Health Activation Index. Mixed-effects linear regression models using repeated measures with anxiety, patient activation, physical and mental health status as outcomes were used to examine their association with smartwatch ownership at baseline.

**Results:**

Ninety-six participants, primarily White with high income and tertiary education, were randomized to receive a study smartwatch-smartphone dyad. Twenty-four (25%) participants previously owned a smartwatch. Compared to those who did not previously own a smartwatch, smartwatch owners reported significant greater increase in their self-reported physical health (*β* = 5.07, *P* < 0.05), no differences in anxiety (*β* = 0.92, *P* = 0.33), mental health (*β* = −2.42, *P* = 0.16), or patient activation (*β* = 1.86, *P* = 0.54).

**Conclusions:**

Participants who own a smartwatch at baseline reported a greater positive change in self-reported physical health, but not in anxiety, patient activation, or self-reported mental health over the study period.

## Introduction

Atrial fibrillation (AF) is associated with a more than 3-fold higher stroke risk among US adults (1). An estimated 1 out of 5 patients who suffer a stroke ultimately have AF diagnosed (2). Stroke has significant adverse effects on patients' actions and quality of life (3, 4). Timely diagnosis of AF and subsequent initiation of anticoagulation therapy can prevent incident and recurrent strokes among older adults (2, 5). Prompt diagnosis of AF, however, remains challenging because of the often clinically silent and intermittent nature of this arrhythmia (2).

Extended ECG monitoring is often utilized in stroke survivors to capture this rhythm abnormality (5). Traditional methods to detect AF such as Holter monitors or implantable loop recorders are limited by their relative low diagnostic yield and invasive nature, respectively (6–8). The development of algorithms using photoplethysmography (PPG) recordings from smartwatches has been shown to be an accurate way to detect heart rhythm abnormalities (9–15). Recently, several wearable devices have been cleared by the FDA for incident AF detection (10). Smartphone and smartwatch ownership and familiarity among US adults over the age of 50 has steadily increased, with about 42% reporting almost daily use (16). As reported by Nuvvula et al. in the NexUS-Heart study, which included 327 AF and 895 older adults at risk for AF, around a third of participants in both groups reported owning a commercial wearable device and the majority had used one for longer than a year (17). About 46% of participants with AF and prior smartwatch ownership used these devices to share information from commercial wearables with their medical team (17). Additionally, a randomized trial of healthy young adults by Yen showed that use of wearables might have positive effects on physical health and quality of life (QoL) (18). A study by Filippaios et al. including older populations suggest a more complex relationship between smart device use and QoL, including potential adverse effects of rhythm alert notifications from a smartwatch on QoL (19).

Familiarity with technology used for AF detection and higher technology engagement at baseline may influence users' experiences with smartphones and wearables. For such devices to be deployed clinically in high-risk older populations, more information is needed. Using data from the randomized trial of older stroke survivors randomized to receive a smartwatch-smartphone dyad vs. conventional monitoring, we examined the potential influence of baseline smartwatch ownership on changes in self-reported anxiety, patient engagement, and health-related quality of life.

## Methods

### Study population

We analyzed data from the Pulsewatch study (NCT03761394) (12), a prospective, randomized controlled, multiphase study to assess the accuracy, feasibility, and compliance of a smartphone-smartwatch dyad for AF detection in patients who suffered from a stroke or transient ischemic attack (TIA). Individuals who meet the criteria to be included in the study were (1) more than 50 years of age; (2) had a history of stroke or TIA, and (3) were able to travel to the clinic at the University of Massachusetts Memorial Medical Center. Those with a serious physical illness that prevents interactions with the smart device, a contraindication for anticoagulation treatment, an incapacity to sign consent, an inability to read and write in English, an unwillingness to complete all study procedures, or plans to move from the area during the study period were excluded (12).

### Study design

Phase I of the Pulsewatch study focused on accuracy and usability of the Pulsewatch system, whereas Phase II focused on adherence to smartwatch prescription for AF detection. At baseline, participants completed a questionnaire assessing their demographic and psychosocial factors. In Phase I, participants were randomized 3:1 into intervention or control groups. Participants in the intervention group were provided with a smartwatch (either Samsung Gear S3 or Galaxy Watch 3) and a Samsung smartphone with our Pulsewatch apps downloaded on them. Participants were asked to wear watches continuously and charge them daily over a 14-day period. In addition, they were given gold-standard FDA-approved Cardiac Insight Cardea Solo cardiac patch monitors (Seattle, WA) and they were asked to wear the patches continuously over a 14-day period. Participants in the control group were provided with only the cardiac patch monitor. At the end of the initial 14-day study period, participants completed a follow-up questionnaire and were once again randomized into either intervention or control groups (1:1). Participants in the intervention group were offered continuous use of the Pulsewatch system (Samsung smartwatch + Samsung Smartphone with study apps) for 30 days, as well as an FDA-approved AliveCor KardiaMobile device (Mountain View, CA) for AF detection, whereas participants from the control group did not receive any devices. University of Massachusetts Chan Medical School Institutional Review Board approved the study protocols (H00016067).

### Self-reported outcomes

Participant information, including demographics, medical history, and psychosocial characteristics was collected by trained research staff (12). All participants completed validated study questionnaires at baseline, 14 days, and 44 days (if randomized to long-term use) to assess various psychosocial measures and health behaviors. We used the Generalized Anxiety Disorder-7 scale (GAD-7), a standardized 7-item questionnaire to assess generalized anxiety (20). The GAD-7 scale was scored from 0 to 3 with overall assessment scores ranging in severity from 0 to 21 (20). Higher scores of GAD-7 indicate higher anxiety levels (20). The Physical Component Summary (PCS) and Mental Component Summary (MCS) of the 12-Item validated Short Form Survey (SF-12) was administered to evaluate physical and mental health-related quality of life, respectively (21). With scores ranging from 0 to 100, higher SF-12 score demonstrates a better quality of life and general health perception (21). The Consumer participants' ability and willingness to manage his or her own health (22). App usability was captured through the Mobile Application Rating Scale (MARS) App Classification.

### Statistical analysis

In this analysis, we included participants who received the smartwatch/smartphone dyad across both phases of the RCT and grouped them into those who owned a smartwatch at baseline vs. those who did not. Differences in baseline characteristics by smartwatch ownership were examined using Fisher's Exact Test and Wilcox Signed Rank Test for categorical and continuous variables respectively. Longitudinal mixed-effects linear regression was used to examine the relationship between psychosocial changes over time and smartwatch ownership prior to enrollment. Due to modest sample size, we did not adjust for any confounding variables. The psychosocial changes were quantified by the standard questionnaires completed by participants at baseline, 14 days, and 44 days. All statistical analyses were completed using SAS 9.4 (SAS Institute Inc. Cary, NC, USA).

## Results

A total of 96 participants were randomized to receive a smartwatch-smartphone dyad for AF detection and completed 45 days of follow-up. The mean age of the participants was 65 ± 9 years, forty-one participants were women (43%), most of the study population was white (88%), over half had completed at least a bachelor's degree, and 68% were married (Table 1). Twenty-four participants owned smartwatches at baseline, whereas 72 participants did not report ownership of a smartwatch. We did not find statistically significant differences in the characteristics of those who owned smartwatches and those who did not with regards to demographic, medical history, and psychosocial characteristics at baseline (Tables 1, 2).

**Table 1:** Baseline demographics and medical history of participants stratified by smartwatch ownership.

Characteristics	Smartwatch ownership at baseline		
	*n *= 96		
Socio-demographics	Yes	No	*P*-value
	*n* = 24	*n* = 72	
Age, mean, years (SD)	63.3 (9.4)	65.0 (8.9)	0.45
Male sex (%)	14 (58.3)	41 (56.9)	0.91
Female sex (%)	10 (41.7)	31 (43.0)	
Race (%)			
White	21 (87.5)	63 (87.5)	0.68
Others	3 (3.1)	9 (3.1)	
Married/Living as married (%)	18 (75.0)	47 (66.2)	0.83
Non-married (%)	6 (6.3)	24 (8.5)	
Education (%)			
Less than high school	0 (0.0)	4 (5.6)	0.11
High school degree or some college	7 (9.72)	36 (16.9)	
College degree	6 (25.00)	15 (21.1)	
Post-graduate studies/degree	11 (22.9)	16 (11.3)	
Income (%)			
Less than 50,000$ annually	6 (6.3)	26 (10.0)	0.11
50,000–99,999$ annually	7 (18.8)	22 (16.9)	
Over 100,000$ annually	11 (22.9)	17 (13.1)	
Physiologic parameters			
BMI, median (IQR)	28.6 (26.3,32.5)	27.9 (26.1,30.9)	0.34
Systolic BP, median (IQR)	132 (122,142)	130.5 (117,141)	0.72
Diastolic BP, median (IQR)	76 (71,83)	77.5 (71,82)	0.63
HR, median (IQR)	67.5 (61,82)	75.0 (61,85)	0.42
Past Medical History (%)			
Vascular Disease	7 (29.2)	17 (23.6)	0.59
Valvular Disease	4 (16.7)	6 (8.3)	0.26
Diabetes Mellitus	3 (12.5)	22 (30.6)	0.11
COPD	1 (4.2)	9 (12.5)	0.44
Renal disease	1 (4.2)	8 (11.1)	0.44
Major bleeding event or predisposition to bleeding	1 (4.2)	5 (6.9)	1.00
Congestive Heart Failure	1 (4.2)	6 (8.3)	0.68
Essential Hypertension	18 (75.0)	55 (76.4)	1.00
Obstructive Sleep Apnea	8 (33.3)	17 (23.6)	0.42
Prior myocardial infarction	5 (20.8)	12 (16.7)	0.76
Hyperlipidemia	19 (79.2)	63 (87.5)	0.33
Stroke History (%)			
Stroke	18 (75.0)	59 (81.9)	0.56
TIA	8 (33.3)	21 (29.2)	0.80
Residual Neurologic Deficits	5 (20.8)	27 (37.5)	0.27
Medication use (%)			
Anticoagulants	4 (16.7)	8 (11.1)	0.49
Antiplatelets	22 (91.7)	61 (84.7)	0.51
Antihypertensives	13 (54.2)	43 (59.7)	0.64
Anti-arrhythmic medications	2 (8.3)	0 (0.0)	0.06
Calcium blockers	4 (16.7)	14 (19.4)	1.00
Beta blockers	8 (33.3)	34 (47.2)	0.34
Statins	24 (100.0)	64 (88.9)	0.19

BMI, body mass index; BP, blood pressure; COPD, chronic obstructive pulmonary disease; HR, heart rate.

**Table 2:** Baseline psychosocial characteristics of participants stratified by smartwatch ownership.

Characteristics	Smartwatch ownership at baseline		
	*n* = 96		
Psychosocial characteristics (%)	Yes	No	*P*-value
	*n* = 24	*N* = 72	
Cognitive impairment	4 (16.7)	25 (35.2)	0.12
Social isolation	4 (16.7)	7 (9.7)	0.46
>8 alcoholic drinks per week	24 (100.0)	72 (100.0)	
Depressive symptoms	8 (33.3)	36 (50.0)	0.24
Minimal	16 (66.7)	36 (50.0)	0.22
Mild	5 (20.8)	26 (36.1)	
Moderate	1 (4.2)	7 (9.7)	
Moderately severe	1 (4.2)	3 (4.2)	
Severe	1 (4.2)	0 (0.0)	
Anxiety Symptoms	8 (33.3)	24 (33.8)	0.97
None	16 (66.7)	47 (66.2)	0.43
Mild	4 (16.7)	18 (25.4)	
Moderate	3 (12.5)	5 (7.0)	
Severe	1 (4.2)	1 (1.4)	
Patient activation			
Low	6 (25.0)	27 (38.6)	0.11
Moderate	16 (66.7)	29 (41.4)	
High	2 (8.3)	14 (20.0)	
Technology engagement (%)			
App use frequency			
Daily	21 (87.5)	38 (61.3)	0.10
A few days a week	1 (4.2)	10 (16.1)	
At least once a week	0 (0.0)	7 (11.3)	
Less than once a week	1 (4.2)	1 (1.6)	
Once a month	1 (4.2)	2 (3.2)	
Never	0 (0.0)	4 (6.5)	

Over the 44 day study period, we observed that self-reported physical health (SF-12 PCS, *β* = 5.07, *P *< 0.05) increased over time in participants who previously owned smartwatches as compared with those who did not, while anxiety (GAD-7, *β* = 0.87, *P* = 0.29), self-reported mental health (SF-12 MCS, *β* = −2.42, *P* = 0.16), and patient activation (CHAI, *β* = 1.86, *P* = 0.54) were not different (Table 3).

**Table 3:** Psychosocial changes after smartwatch prescription in participants with prior smartwatch ownership compared to those without.

	β−Estimate (SE)^a,b^	*P*-value
Generalized Anxiety Disorder-7 score	0.92 (0.83)	0.27
Physical Health Short Form-12 survey	5.12 (2.14)	**<0**.**05**
Mental Health Short Form-12 survey	−2.38 (1.71)	0.17
Consumer Health Activation Index score	1.90 (3.00)	0.53

^a^ A higher GAD7 indicates poorer psychosocial outcome (anxiety), while higher CHAI or SF-12 indicates better psychosocial outcomes (self-reported health).

^b^ Controlling for AF detected on smartwatch.

The bold value indicates statistical significant difference in self-reported physical health in participants with prior smartphone ownership compared to those without.

## Discussion

In this multiphase RCT of older stroke survivors randomized to receive a smartwatch for AF detection, we found that participants who previously owned smartwatches reported increased perceived physical health over the study period. No effect was detected on anxiety level, mental health status, or activation towards self-care.

Participants who previously owned smartwatches appear to report better overall physical health as measured by the SF-12 questionnaire after being prescribed a smartwatch for AF detection. It could be that familiarity with wearable devices empowered participants to monitor and engage in physical activities that leads to better perceived physical health. This change in perceived physical health is perhaps not mediated by anxiety or mental health as we did not observe differences reported in GAD-7 or SF12 MCS score over the study period with respect to prior smartwatch ownership. Consistent with our findings, Yen's analysis on another RCT comparing the use of smartwatches to traditional mobile applications in healthy young adults demonstrated better physical quality of life (QOL) in the smartwatch arm (Table 4) (18). Our results are further supported by findings of Macridis et al. demonstrating that consumer wearable ownership and use among Canadian adults is correlated with meeting physical activity guidelines (23). Additionally, awareness of how these wearable devices operate by these smartwatch owners also increased their comfort with using the study's devices which may have led to higher perceived physical health activity and quality of life.

**Table 4:** Comparison of the current study design with other smartwatch atrial fibrillation (AF) and heart arrhythmia detection studies.

Study	Study Population	Intervention	Outcomes	Findings
Mensah Otabil et al.	Participants were 50 years or older and had survived a stroke or TIA	Participants in the intervention group received smartwatch-smartphone dyad in addition to the cardiac patch monitors to wear for 14 days and use dyad with AliveCor Kardia device for an additional 30 days. Control groups received the patch monitors for the 14-day period and responded to exit questions at the end of the 44 days.	GAD, SF-12 PCS and MCS were measured at baseline, 14 days, and 44 days between participants who previously owned smartwatches vs. those who did not.	Participants who previously owned smartwatches had a significant increase in SF-12 PCS than those who did not own a smartwatch. No differences were noted in terms of anxiety, SF-12 MCS, and patient activation in both groups.
Nuvvula et al. (17)	Participants were 18 years or older and had AF or at risk of developing AF (65 years old and older and CHA_2_DS_2_-VASc stroke risk of >2)	Participants were provided survey questionnaires to determine their behavior around telehealth and smartwatch use.	Health technology use and telemedicine engagement surveys	Participants with AF were more likely to share their wearable-device-derived health data (such as heart rate and rhythm) with providers in comparison to those at risk of AF.
Yen (18)	Participants were between the ages of 20–65 years old and had smartphones with the ability to download apps.	Participants in the experimental groups received either a smart bracelet or a smartwatch which connected to a mobile app on their smartphone. Control group only used mobile apps.	Health Promotion Lifestyle Profile (HPLP-S). World Health Organization Quality-of-Life Scale (WHOQOL-BREF) at baseline and at the 3-month study endpoint.	Participants in the intervention group exercised more, managed stress better, lived healthier lives, and had better physical and mental quality of life in comparison to the control group with only mobile apps.
Filippaios et al. (19)	Participants were 50 years or older and had survived a stroke or TIA.	Participants in the intervention group received smartwatch-smartphone dyad in addition to the cardiac patch monitors to wear for 14 days and use dyad with AliveCor Kardia device for an additional 30 days. Control groups received the patch monitors for the 14-day period and responded to exit questions at the end of the 44 days.	GAD-7, SF-12, CHAI were measured at various time points during study and compared between those with AF alerts and those without	AF alerts from wearable devices such as smartwatches are unlikely to cause significant anxiety among older groups but may lead to a lower perception of their health.
Macridis et al. (23)	Participants were adults ≥18 years from Alberta, Canada, and were recruited through random-digit dialing	Random-digit dialing with computer-assisted telephone interviewing	Questionnaires to assess demographic and health behavior variables to assess physical activity tracking (PAT) devices ownership and usage	PAT ownership and use associated with female, < 60 years of age, having a post-secondary education, meeting physical activity guidelines, and being overweight/obese.

Despite the positive perception on physical health by prior owners of a smartwatch, their mental health status was not significantly different from that of participants who did not own a smartwatch. The mental health component of SF-12 questionnaire is a useful tool for monitoring mental status for targeted treatment (20). As a new treatment with a new device, it is important to evaluate whether such intervention would affect that individual's mental health. Continuous, instant monitoring was found to bring unnecessary anxiety to people who were using wearable devices for health purposes (23). Studies have shown that a repeated alert and notification system in wearable devices can trigger anxiety and other mental health issues in older populations. In our study, however, we used two validated assessments to evaluate psychological status change: mental health component of SF-12 and GAD-7 score. Both showed no difference between participants who previously owned a smart device and those who did not. Our results suggest that clinicians should not shy from prescribing smartwatch for AF screening in those with less exposure to digital technology as we find no significant differences in change in self-reported anxiety and mental health with respect to prior ownership of smartwatch.

Our work provides evidence of feasibility in using smartwatch devices in an elderly population and showed the potential positive health effects of their use. In our study, participants with prior use of smartwatches, reported higher perceived physical health when prescribed smartwatches for AF detection post stroke, in comparison to participants who had never used a smartwatch before. In this context, it might be helpful for providers to consider familiarity and baseline technology engagement of participants, when applying modern technologies in the routine monitoring and screening for atrial fibrillation. Prior ownership of smartwatches had no effect on changes in anxiety levels, mental health status, or participant activation during the time of smartwatch monitoring. Albeit further studies are necessary to investigate this association, a smartwatch-naïve population, adequately trained and educated by their medical teams, can likely benefit from rhythm monitoring via smartwatches. Lack of familiarity with smartwatch technology at baseline did not seem to have a negative impact on anxiety, self-perceived mental health and patient activation while using smartwatches for rhythm monitoring.

### Strengths and limitations

Our cohort has several strengths. First, the cohort's participants had rigorous assessments of sociodemographic, clinical, and psychosocial characteristics. We utilized standardized questionnaires including GAD-7, CHAI, and SF-12 at several time-points to examine changes in, anxiety, patient activation, and health-related quality of life among participants in a randomized trial, increasing the generalizability and potential reproducibility of our study findings. However, participants were recruited from a single tertiary medical center in central Massachusetts. Most of our participants are white stroke survivors with some level of college education; this may limit generalization of our findings to other populations. Further, this is a *post-hoc* secondary analysis of a modestly sized cohort and may lack the power to detect potential changes of self-reported outcomes over a relatively short period of observation.

## Conclusion

In our study of elderly stroke survivors randomized to receive a smartwatch and smartphone for AF monitoring and followed for 45 days, participants who owned a smartwatch at baseline reported better physical health than participants who did not own smartwatches. No differences were noted between smartwatch owners and non-owners as it relates to anxiety, mental health status and activation. Our study suggests that technology familiarity at baseline strongly influences patient experience and quality of life among those prescribed technologies for AF monitoring. Large studies are needed to further understand how best to improve health and well-being among older adults who are not familiar with digital devices. Studies evaluating mobile health approaches to AF detection should assess baseline technology familiarity and ownership.

## Data Availability

The raw data supporting the conclusions of this article will be made available by the authors, without undue reservation.
